# Pharmacological Properties of a Traditional Korean Formula Bojungchiseup-tang on 3T3-L1 Preadipocytes and High-Fat Diet-Induced Obesity Mouse Model

**DOI:** 10.1155/2020/8851010

**Published:** 2020-11-29

**Authors:** Yea-Jin Park, Dong-Wook Seo, Tae-Young Gil, Divina C. Cominguez, Hwan Lee, Dong-Sung Lee, Insik Han, Hyo-Jin An

**Affiliations:** ^1^Department of Pharmacology, College of Korean Medicine, Sangji University, Wonju, Gangwon-do 26339, Republic of Korea; ^2^College of Pharmacy, Chosun University, 309 Pilmun-daero, Dong-gu, Gwangju 61452, Republic of Korea; ^3^Department of Internal Medicine of Korean Medicine, Oriental Medicine Hospital of Sang-ji University, Republic of Korea

## Abstract

The global obesity epidemic has nearly doubled since 1980, and this increasing prevalence is threatening public health. It has been reported that natural products could contain potential functional ingredients that may assist in preventing obesity. Bojungchiseub-tang (BJT), mentioned in the *Donguibogam* as an herbal medication for the treatment of edema, a symptom of obesity, consists of eleven medicinal herbs. However, the pharmacological activity of BJT has not been investigated. The present study was designed to investigate the putative effect of BJT on the adipogenesis of 3T3-L1 cells and the weight gain of high-fat diet (HFD-) fed C57BL/6 mice. Oil Red O staining was conducted to examine the amount of lipids in 3T3-L1 adipocytes. Male C57BL/6 mice were divided into three groups: standard diet group (control, CON), 45% HFD group (HFD), and HFD supplemented with 10% of BJT (BJT). The expression levels of genes and proteins related to adipogenesis in cells, WAT, and liver were analyzed by quantitative real-time polymerase chain reaction (qRT-PCR) and western blot, respectively. We found that BJT treatment significantly decreased the protein and mRNA levels of peroxisome proliferator-activated receptor *γ* (PPAR*γ*), CCAAT/enhancer-binding protein *α* (C/EBP*α*), and sterol regulatory element-binding protein 1 (SREBP1) in a dose-dependent manner in differentiated 3T3-L1 cells. Similar to the results of the *in vitro* experiment, BJT suppressed HFD-induced weight gain in an obese mouse model. In addition, BJT effectively reduced the HFD-induced epididymal adipose tissue weight/body weight index. BJT also downregulated the mRNA levels of *PPARγ*, *C/EBPα*, and *SREBP1* in the epididymal adipose and liver tissue of HFD-fed obese mice. These findings suggest that BJT induces weight loss by affecting adipogenic transcription factors.

## 1. Introduction

Obesity has been increasing in epidemic proportions in the United States and most of the Westernized world [1]. Obesity leads to various metabolic diseases, such as hypertension, nonalcoholic fatty liver disease, atherosclerosis, type 2 diabetes, and cardiovascular diseases [2]. Obesity is mainly caused by a chronic imbalance between energy intake and expenditure, resulting in increased adipose tissue mass [3]. White adipose tissue is a multifactorial organ conducting intricate metabolic functions under physiological conditions; however, during obesity, it may become severely dysfunctional and fail to appropriately expand and store the surplus energy [4]. Fat mass is locally determined via the function of white adipocytes and the modulation of white adipogenesis, the process of preadipocyte differentiation into adipocytes [5]. Increased adipocyte size is associated with increased systemic insulin resistance, while small adipocytes are correlated with decreased metabolic health and diabetes [6]. Additionally, obesity directly contributes to an increase in hepatic triglyceride accumulation, which is related to the progression of nonalcoholic fatty liver disease [7]. A number of genes have been shown to be involved in the development of obesity, including peroxisome proliferator-activated receptor *γ* (PPAR*γ*), CCAAT/enhancer-binding protein *α* (C/EBP*α*), and sterol regulatory element-binding protein 1c (SREBP1c) [8]. SREBP1c stimulates the expression of PPAR*γ*, C/EBP*α*, and several other lipogenic enzyme products [9].

Natural products such as herbs, fruits, and vegetables have recently been shown to have inhibitory effects on adipocyte lipid accumulation through the induction of apoptosis, cell cycle arrest, and transcription factors. Consequently, they could be potential functional ingredients in preventing obesity, with high expectations regarding their efficacy, safety, and long-term effects [10]. Bojungchiseub-tang (BJT) is mentioned in the *Donguibogam* as an herbal medication that has been used to treat signs of edema, dampness-phlegm, and kidney failure [11]. Individuals with a phlegm-dampness constitution have a much higher risk of obesity than those with a balanced constitution [12]; edema is also a common finding in obesity [13]. BJT consists of 11 medicinal herbs, all with proven effects on health. *Panax ginseng* C.A. Meyer and *Atractylodes macrocephala* Koidzumi are known to prevent obesity and dyslipidemia in high-fat diet (HFD-) fed castrated mice [14, 15]. *Poria cocos* Wolf exerted a protective effect against hepatic steatosis in HFD obese mice [16]. The compound MDG-1 from *Ophiopogon japonicas* shows potent hypoglycemic and weight control effects in mice [17]. Decursin, present in *Angelica gigas* Nakai, has an antiadipogenic effect in adipose-derived stem cells isolated from human visceral adipose tissue [18]. *Akebia quinata* extract also has antiobesity and hypolipidemic effects in HFD-fed mice [19]. *Scutellaria baicalensis* has favorable effects on hyperglycemia, glucose tolerance, hyperinsulinemia, and hypertriglyceridemia in mice [20]. In the present study, we investigated the putative effect of BJT on the adipogenesis of 3T3-L1 cells, as well as on the weight gain of HFD-fed C57BL/6 mice.

## 2. Materials and Methods

### 2.1. Chemicals and Reagents

3-Isobutyl-1-methylxanthine (IBMX), dexamethasone (DEX), insulin, and Oil Red O powder were purchased from Sigma-Aldrich Co. LLC (St. Louis, MO, USA). Dulbecco's modified Eagle's medium (DMEM), bovine serum (BS), fetal bovine serum, and antibiotic-antimycotic (ABAM) were obtained from Life Technologies, Inc. (Grand Island, NY, USA). A 45% HFD was acquired from research diets (New Brunswick, NJ, USA). PPAR*γ*, C/EBP*α*, SREBP1, and glyceraldehyde-3-phosphate dehydrogenase (GAPDH) oligonucleotide primers were purchased from Bioneer Corporation (Daejeon, Republic of Korea), and SYBR Premix Ex Taq was purchased from Takara Bio Inc. (Otsu, Japan). Antibodies against PPAR*γ* (cat. No. sc-7273), C/EBP*α* (cat. No. sc-9314), SREBP1 (cat. No. sc-13551), and *β*-actin (cat. No. sc-81178) were purchased from Santa Cruz Biotechnology, Inc. (Dallas, TX, USA). Horseradish peroxidase-conjugated secondary antibodies were obtained from Jackson ImmunoResearch Laboratories, Inc. (West Grove, PA, USA).

### 2.2. Preparation of BJT

BJT contained *Panax ginseng* C. A. Meyer (4 g), *Atractylodes macrocephala* Koidzumi (4 g), *Atractylodes chinensis* Koidzumi (2.8 g), *Citrus unshiu* Markovich (2.8 g), *Poria cocos* Wolf (2.8 g), *Liriope platyphylla* Wang et Tang (2.8 g), *Akebia quinata* Decaisne (2.8 g), *Angelica gigas* Nakai (2.8 g), *Scutellaria baicalensis* Georgi (2 g), *Magnolia obovata* Thunberg (1.2 g), and *Cimicifuga heracleifolia* Komarov (1.2 g). All 11 herbs were acquired from Nanum Pharmaceutical Company (Seoul, Republic of Korea). The extraction yield of herbs was performed as previously described [21]. Herbs were extracted in the water at 99°C for 3 h. The extract was then freeze-dried, and the yield was calculated at 14%. The powder was dissolved in distilled water for subsequent experimentation, and the residual powder was stored at -20°C.

### 2.3. Cell Culture of 3T3-L1 Preadipocytes

3T3-L1 preadipocytes were obtained from the American Type Culture Collection (ATCC, Manassas, VA, USA; CL-173) and were cultured in DMEM containing 10% BS, 1% ABAM, 1 g/l HEPES, and 1.5 g/l sodium bicarbonate. The 3T3-L1 adipocyte differentiation was performed as previously described [22]. To stimulate adipocyte differentiation, cells were seeded at a density of 2 × 10^5^ per well into 6-well plates to confluence (day 0). Then, cells were differentiated with an MDI medium containing 0.5 mM IBMX, 1 *μ*M DEX, and 1 *μ*g/ml insulin in the culture medium (day 2). The cells were also differentiated in a culture medium containing 1 *μ*g/ml insulin (day 4). The culture medium was changed every 2 days, until days 6–8.

### 2.4. Cell Viability Assay

Cell viability was performed was previously described [22]. 3 T3-L1 preadipocytes (1 × 10^4^ cells per well) were seeded on 96-well plates at density 1 × 10^4^ cells per well. After 24 h, cells were treated with different concentrations of BJT for 48 h. The 3-(4,5-dimethylthiazol-2-yl)-2,5-diphenyl tetrazoliumbromide (MTT) solution (5 mg/ml) was treated and the cells were incubated at 37°C for 4 h. After discarding the supernatant, 100 *μ*l of dimethyl sulfoxide was added to dissolve formazan crystals, and the MTT-formazan product was measured using an Epoch® microvolume spectrophotometer (Bio Tek Instruments Inc., Winooski, VT, USA) at 570 nm.

### 2.5. Oil Red O Staining of 3T3-L1 Adipocytes

The Oil Red O staining in 3 T3-L1 adipocytes was performed as previously described [22]. 3 T3-L1 preadipocytes were cultured with or without differentiation conditions in the presence or absence of indicated concentrations of BJT. After 8 days of differentiation, cells were washed three times with phosphate-buffered saline (PBS) and fixed with 10% formaldehyde in PBS at 25°C for 1 h. After fixation, cells were washed three times with distilled water and then stained with Oil Red O working solution (3 mg/ml ORO in 60% isopropanol) at 25°C for 2 h. Cells were rinsed three times with distilled water and photographed with an Olympus SZX10 microscope (Tokyo, Japan). The Oil Red O dye was dissolved by isopropanol and measured with an Epoch® microvolume spectrophotometer at 520 nm.

### 2.6. Western Blot Analysis

Western blot analysis was performed as previously described [23]. The liver tissue and 3T3-L1 cells were homogenized with PRO-PREP™ protein extraction solution (Intron Biotechnology, Seoul, Republic of Korea). Equal amounts (15–30 *μ*g) of protein samples were separated on a sodium dodecyl sulfate-polyacrylamide gel and then transferred onto a polyvinylidene fluoride membrane. Membranes were incubated overnight with primary antibody PPAR*γ*, C/EBP*α*, and SREBP1 and incubated with horseradish peroxidase-conjugated secondary antibody for 2 h. The blots were again washed three times with tris buffered saline with tween 20 and then visualized by enhanced chemiluminescence using Amersham™ Imager 680 (GE Healthcare Bio-Sciences AB, Sweden).

### 2.7. Quantitative Reverse-Transcription Polymerase Chain Reaction (qRT-PCR) Analysis

The qRT-PCR analysis was performed as previously described [23]. In brief, the liver, epididymal adipose tissue, and 3T3-L1 cells were homogenized, and total RNA was isolated using the Easy-Blue® reagent according to the manufacturer's instructions (Intron Biotechnology; Seongnam, Republic of Korea). Total RNA was converted to cDNA using a high-capacity cDNA reverse transcription kit (Applied Biosystems; Foster City, CA, USA) and thermocycler (Gene Amp PCR system 9700; Applied Biosystems) with the following program: initiation for 10 min at 25°C, followed by incubation at 50°C for 90 min and at 85°C for 5 min. qPCR analysis was conducted using a StepOnePlus Real-time PCR system (Applied Biosystems). Gene expression was determined according to the comparative threshold cycle (Ct) method. GAPDH was used as an internal control. Sequences of mouse oligonucleotide primers are presented in Table 1.

### 2.8. Experimental Animals

Eight-week-old male C57BL/6N mice (specific-pathogen-free (SPF) grade, 18–20 mg) were obtained from Daehan Biolink (Daejeon, Republic of Korea) and maintained at modified conditions (22 ± 2°C and 55 ± 9% humidity, 12 h light/dark cycle). Prior to the initiation of the experiment, the mice were acclimatized to their environment for 1 week. Thereafter, mice were weighed and divided into three groups (*n* = 6 per group) as follows: standard diet group (control, CON), 45% HFD group (HFD), and HFD supplemented with 10% of BJT (BJT). The animals were subjected to experimentation for 11 weeks, during which period all mice had ad libitum access to food and water. Mouse body weight and food intake were measured weekly for 11 weeks. At the end of the experiment, mice were anesthetized with Zoletil 50 (20 mg/kg) by intraperitoneal injection according to the manufacturer's instructions after 12 h of fasting (only water was provided). Animals were euthanized by cervical dislocation. Liver and adipose tissue were excised, rinsed with PBS, immersed into liquid nitrogen, and stored at −80°C until further experimentation. All experiments were conducted with approval from the Ethical Committee for Animal Care and the Use of Laboratory Animal of Sangji University (reg.no. 2018-21).

### 2.9. Biochemical Analysis

Serum biochemical analysis was performed as previously described [24]. Blood was collected and immediately centrifuged (1000 × *g* for 20 min at 4°C) to obtain the plasma. The levels of triglycerides (TG), total cholesterol (TC), aspartate aminotransferase (AST), alanine aminotransferase (ALT), and blood urea nitrogen (BUN) in the plasma were measured using commercial kits (Asan Pharmaceutical. Co. Ltd., Republic of Korea). The concentration of creatinine in the plasma was determined using a commercial kit (BioAssay Systems, Hayward, CA, USA). All biochemical assay was conducted according to the manufacturer's instructions.

### 2.10. Histological Examination

Histological analyses of the liver and adipose tissue were performed as previously described [24]. Liver and epididymal adipose tissue from representative mice were fixed in 10% formalin, embedded into the paraffin, and cut into 5 *μ*m sections. The sections were then used for hematoxylin/eosin staining. The stained liver sections were observed for the examination of lipid droplets. The stained adipose tissue sections were used to measure the size of adipocytes. All observations were performed using an Olympus SZX10 microscope.

### 2.11. Statistical Analysis

Each result is represented as the mean ± standard deviation of triplicate experiments. Statistical analysis was performed using SPSS version 19.0 (International Business Machines, Armonk, NY, USA). Statistical significance was determined using analysis of variance and Dunnett's post hoc test. *P* values of less than 0.05 were considered statistically significant amongst the experimented groups.

## 3. Results

### 3.1. BJT Suppressed Adipogenesis in 3T3-L1 Cells

**Figure 1:**
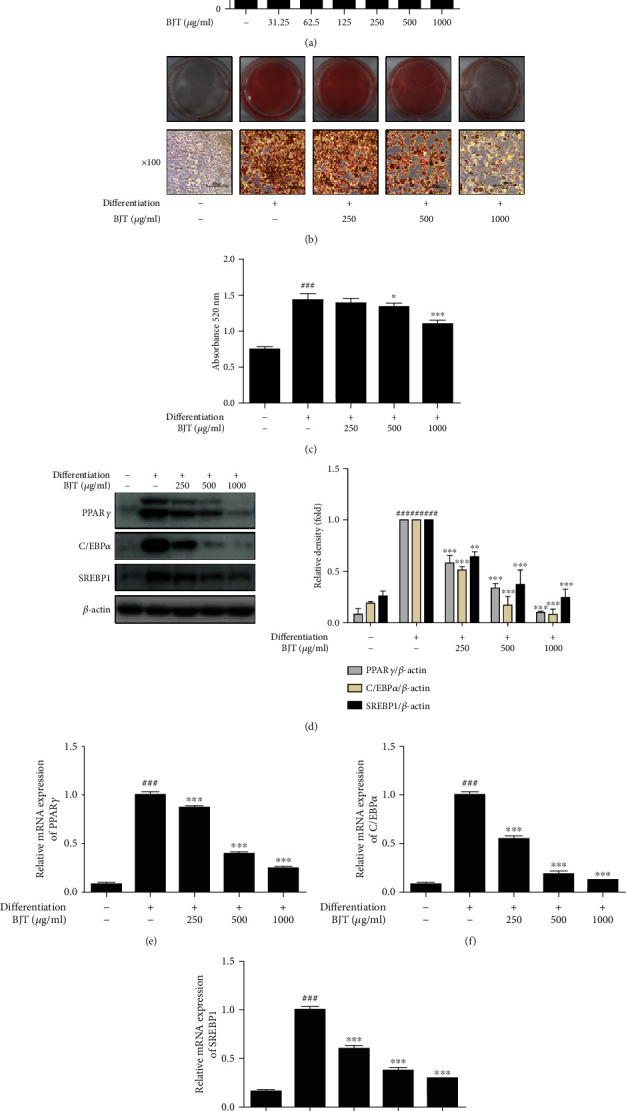
Effect of BJT on adipogenesis of 3T3-L1 cells. Preadipocytes were treated with various concentrations of BJT for 48 h, and their viability was estimated using MTT assay (a). 3T3-L1 cells were stimulated by differentiation medium in the presence or absence of indicated BJT concentrations for 8 days and subjected to ORO staining (b). The stained cells were visualized using a microscope at 100x magnification. Oil Red O was dissolved by isopropanol and measured at 510 nm (c). 3T3-L1 cells were cultured in a differentiation medium in the presence or absence of the indicated concentrations of BJT until day 6. Western blot was performed to determine the protein level of PPAR*γ*, C/EBP*α*, and SRBP1. For clarity, cropped gel images are shown (d). qRT-PCR was performed to determine the mRNA level of PPAR*γ* (e), C/EBP*α* (f), and SRBP1 (g). The values are represented as mean ± S.D. ^###^*P* < 0.001 vs. the nondifferentiation group; ^∗^*P* < 0.05, ^∗∗^*P* < 0.01, and ^∗∗∗^*P* < 0.001 vs. the differentiation group; significances were determined using one-way ANOVA followed by a Dunnett's post hoc test.

MTT assay was used to determine the effect of BJT on cell viability in 3T3-L1 preadipocytes. The results showed that concentrations of 1000 *μ*g/ml and below had no toxic effects (Figure 1(a)). Therefore, concentrations at 250, 500, and 1000 *μ*g/ml were used for further experiments. To estimate lipid accumulation, Oil Red O staining was carried out on day 8 of the differentiation process. BJT decreased the intracellular lipid accumulation in a dose-dependent manner relative to that in the nondifferentiated cells (Figures 1(b) and 1(c)). As expected, the protein expression of PPAR*γ*, C/EBP*α*, and SREBP1 increased relative to that in the nondifferentiation group. However, the BJT treatment markedly attenuated these increases in differentiated 3T3-L1 cells (Figure 1(d)). Consistently, the mRNA levels of *PPARγ*, *C/EBPα*, and *SREBP1* increased in the differentiated cells compared to that in the nondifferentiated cells. BJT treatment significantly decreased the levels of PPAR*γ*, C/EBP*α*, and SREBP1 in 3T3-L1 cells in a dose-dependent manner (Figures 1(e), 1(f), and 1(g)).

### 3.2. BJT Suppressed HFD-Induced Body Weight Gain in C57BL/6N Mice

**Figure 2:**
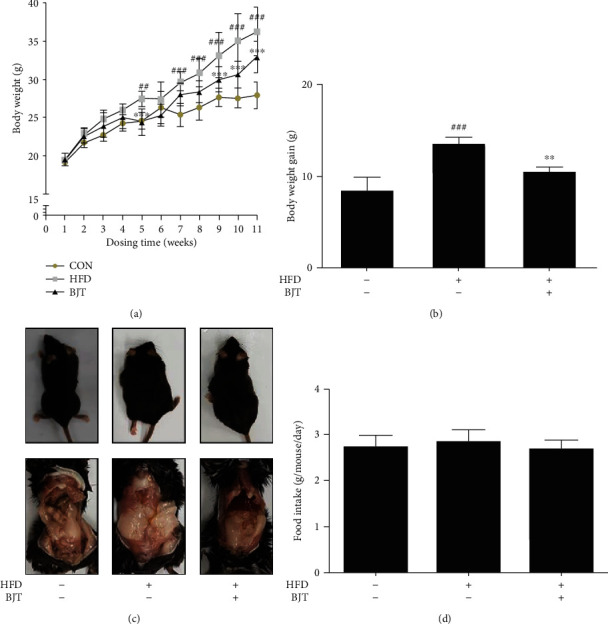
Effect of BJT on body weight and gain weight in HFD-induced obese mice. Mouse body weight was measured weekly during 11 weeks (a), the body weight gain was calculated (b), and mouse bodies were dissected (c, abdominal view). The food intake was measured weekly and calculated (d). The values are represented as mean ± S.D. ^##^*P* < 0.01 and ^###^*P* < 0.001 vs. CON; ^∗∗^*P* < 0.01 and ^∗∗∗^*P* < 0.001 vs. HFD group; significances were determined using two-way ANOVA followed by a Bonferroni post hoc test, and one-way ANOVA followed by a Dunnett's post hoc test.

After 1 week of acclimatization, mice were randomly sorted into three groups, CON, HFD, and BJT. The body weight of the HFD group was significantly more than that of the CON group, while the BJT group had significantly lower body weight than the HFD group (Figure 2(a)). The final average body weight of mice at the end of the experiment was as follows: CON, 27.9 ± 1.76 g; HFD, 36.2 ± 3.16 g; and BJT, 32.9 ± 2.05 g. Furthermore, the weight gain in the HFD group (13.5 ± 2.74 g) was significantly higher than that in the CON group (8.45 ± 1.38 g), but this was significantly inhibited in the BJT group (10.4 ± 1.6 g) (Figure 2(b)). Additionally, the adiposity in the HFD group was more prominent than that in the CON group, while the BJT treatment appeared to block this change relative to that in the HFD group (Figure 2(c)). The food intake was comparable in all three groups (Figure 2(d)). Furthermore, the serum TG and TC levels of the HFD group were significantly higher than those of the CON group, while these levels of the BJT group were significantly lower than those of the HFD group (Table 2).

### 3.3. BJT Suppressed HFD-Induced Lipid Accumulation in Epididymal Adipose Tissue of C57BL/6N Mice

**Figure 3:**
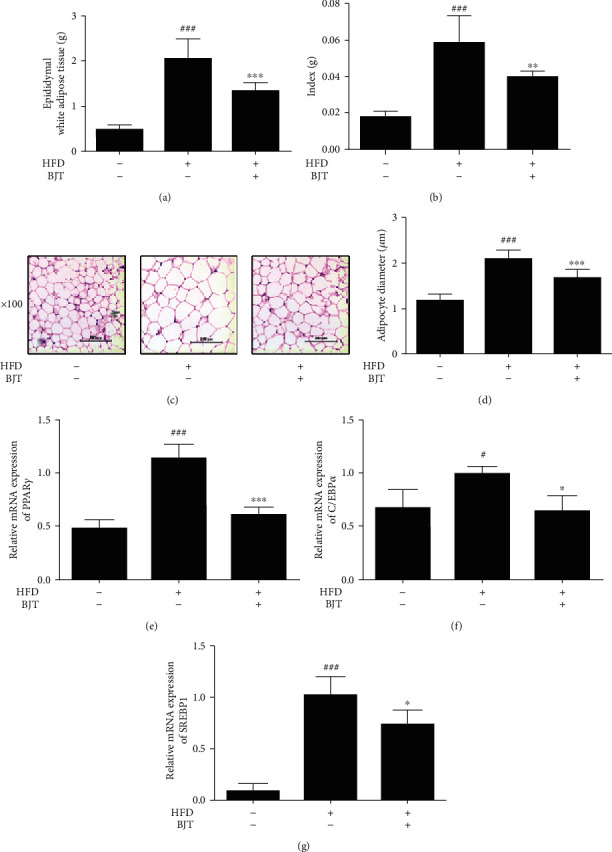
Effect of BJT on the lipid accumulation in epididymal adipose tissue of HFD-induced obese mice. At the end of the experimental period, the weight of epididymal adipose tissue was measured (a) and then divided by the body weight of mice (b). The hematoxylin/eosin staining images are shown at the magnification 100x (c). The average diameter of adipocytes in epididymal adipose tissue of each group (d). qRT-PCR was performed to determine the mRNA level of PPAR*γ* (e), C/EBP*α* (f), and SRBP1 (g). The values are represented as mean ± S.D. ^#^*P* < 0.05 and ^###^*P* < 0.001 vs. CON; ^∗^*P* < 0.05, ^∗∗^*P* < 0.01, and ^∗∗∗^*P* < 0.001 vs. HFD group; significances were determined using one-way ANOVA followed by a Dunnett's post hoc test.

The HFD group showed a significant increase in epididymal adipose tissue weight relative to that in the CON group, while BJT markedly attenuated the increase seen in the HFD group (Figure 3(a)). Likewise, BJT reduced the epididymal adipose tissue weight/body weight index compared to that in the HFD group (Figure 3(b)). Consequently, hematoxylin/eosin staining showed that the size of adipocytes in the HFD group was larger than that in the CON group, and BJT dramatically inhibited the HFD-induced enlargement of epididymal adipocytes (Figures 3(c) and 3(d)). Furthermore, the adipogenic transcription markers *PPARγ*, *SREBP1*, and *C/EBPα* were all dramatically increased by HFD in the epididymal adipose tissue of mice. Importantly, BJT treatment effectively reversed the increases observed in the HFD group (Figures 3(e)–3(g)).

### 3.4. BJT Suppressed HFD-Induced Lipid Accumulation in the Liver Tissue of C57BL/6N Mice

**Figure 4:**
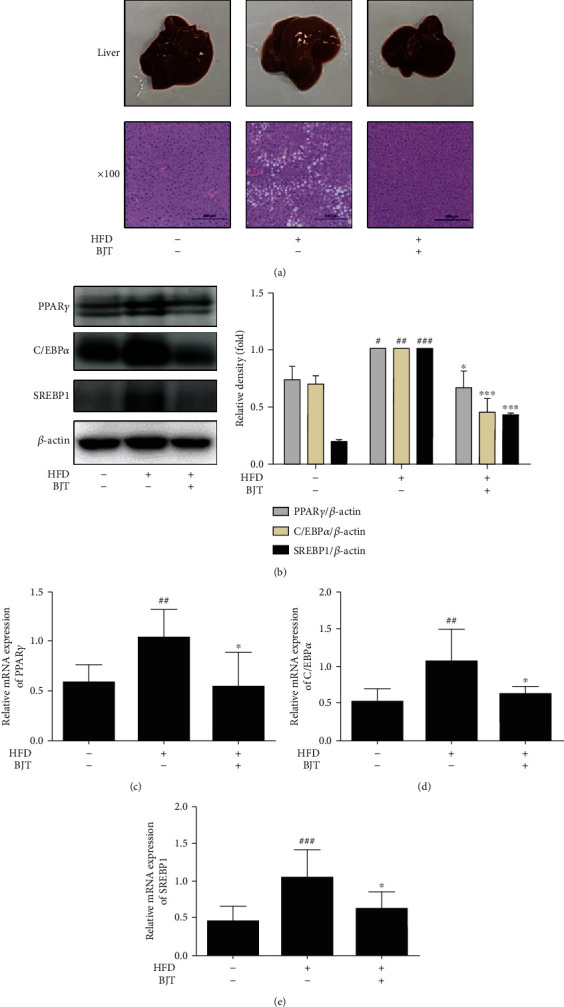
Effect of BJT on the lipid accumulation in liver tissue of HFD-induced obese mice. At the end of the experimental period, the mouse liver was dissected for macroscopic analysis and subjected to hematoxylin/eosin staining (a). Images are shown at the magnification 100x. Western blot was performed to determine the protein level of PPAR*γ*, C/EBP*α*, and SRBP1. For clarity, cropped gel images are shown (b). qRT-PCR was performed to determine the mRNA level of PPAR*γ* (c), C/EBP*α* (d), and SRBP1 (e). The values are represented as mean ± S.D. ^#^*P* < 0.05, ^##^*P* < 0.01, and ^###^*P* < 0.001 vs. CON; ^∗^*P* < 0.05 and ^∗∗∗^*P* < 0.001 vs. HFD group; significances were determined using one-way ANOVA followed by a Dunnett's post hoc test.

To determine the effects of BJT on morphological and histological changes of liver tissue, hematoxylin/eosin staining was performed. The liver tissue in the HFD group was of a lighter color than that in the CON group, whereas BJT treatment reversed this color change. Consistently, HFD increased lipid accumulation compared to that in the CON group in liver tissue, while BJT treatment notably reduced HFD-induced enlargement of lipid droplets in the liver of obese mice (Figure 4(a)). Both the protein and mRNA levels of the adipogenic transcription markers PPAR*γ*, SREBP1, and C/EBP*α* significantly increased in the liver tissue of the HFD group. In contrast, the BJT treatment markedly suppressed these increases in the HFD group (Figures 4(b)–4(e)).

## 4. Discussion

BJT is an herbal medicine to treat symptoms and signs of edema and is widely used in Korean oriental medicine practitioners. Its effect in reducing adipocyte differentiation and adipogenesis has been demonstrated in experiments with 3T3-L1 cells [25]. BJT is composed of eleven herbal plants; among them, five herbs are known to have antiobesity effects. Particularly, ginseng, the main herb of BJT, has been traditionally used in the treatment of most ageing-related diseases including obesity [26]. Ginseng and its active compounds ginsenosides inhibit obesity in several obese animal models [26–31]. In addition, *Atractylodes macrocephala* Koidzumi, another main herb of BJT, prevents diet-induced obesity and glucose intolerance in mice and inhibits adipogenesis in 3T3-L1 cells [15, 32]. Despite its pharmacological studies for the compositional herbs of BJT in treating obesity, the antiobesity effect of BJT has not yet been elucidated. Therefore, we hypothesized that BJT would be effective in treating obesity, and we sought to validate this through *in vivo* model as well as *in vitro* model.

The global obesity epidemic has nearly doubled since 1980, and this increasing prevalence is a threat to public health [33]. Weight gain is the result of an imbalance between total energy intake and total energy expenditure, and it is thought that a substantial and sustained increase of total energy intake over the past 30 years has led to the increase in body weight across the global population [34]. In this study, mice were fed HFD to induce weight gain through an increase in energy intake. BJT has long been used to treat edema and dampness-phlegm [11], and its components have been shown to have antiobesity activities [12–20], but the pharmacological activity of BJT has not yet been investigated. In the present study, we found that BJT suppressed HFD-induced weight gain in an obese mouse model. However, there were no significant changes in hepatic AST and ALT levels, and kidney BUN and creatinine levels in all three groups (Table 2), indicating that BJT reduced body weight without inducing liver and kidney toxicity.

**Figure 5:**
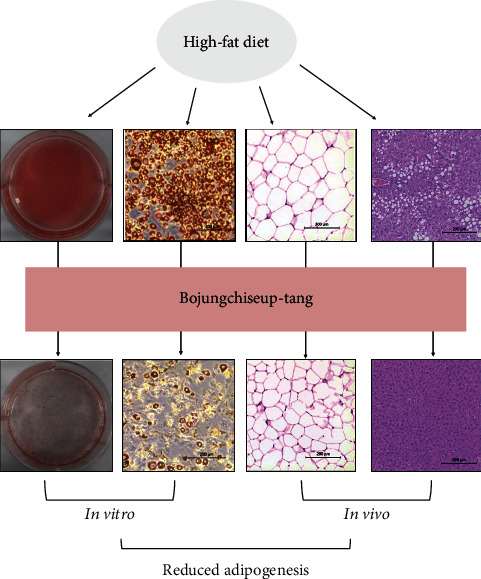
The mechanism of BJT. BJT alleviated lipid accumulation both in the epididymal adipose tissue and liver tissue, as well as *in vitro* model.

Obesity is a multifactorial disorder, characterized by expanding fat mass. BJT treatment significantly reduced the weight of adipose tissue in HFD-induced obesity. Additionally, we examined the effect of BJT on the differentiation of 3T3-L1 adipocytes. 3T3-L1 cells are fibroblast-like preadipocytes, which differentiate into adipocytes, and are commonly used to study adipogenesis [35]. Master transcriptional regulators of adipogenesis, including PPAR*γ*, C/EBP*α*, and SREBP1c, are necessary modulators of target gene expression that are involved in adipocyte differentiation at various stages [36]. C/EBPs consists of six different proteins, C/EBP*α*, C/EBP*β*, C/EBP*γ*, C/EBP*δ*, C/EBP*ε*, and C/EBP*ζ*, that are widely expressed in numerous tissues and regulate many cellular processes, including cell cycle, inflammation, differentiation, metabolism, and cellular proliferation [37, 38]. Among them, C/EBP*α* is expressed in numerous tissues, including in the liver, adipose tissue, skeletal muscle, colon, and prostate, and is mainly observed in terminally differentiated cells [37]. Moreover, C/EBP*α* is expressed during adipocyte differentiation in culture cells at later stages and stays active in mature adipocytes [39]. Herein, BJT decreased the C/EBP*α* levels in 3T3-L1 adipocytes, epididymal adipose tissue, and liver, thus indicating that BJT can induce fat loss by reducing the adipocyte differentiation in the epididymal adipose tissue and liver. C/EBP*α* acts in concert with PPAR*γ* to establish phenotypes of mature adipocytes [40]. The members of the PPAR family of nuclear receptors have vital roles in lipid metabolism [41]. In particular, PPAR*γ* plays a key role in adipogenesis and has been implicated in the pathology of numerous diseases including obesity, type 2 diabetes, and atherosclerosis [42]. Adipocyte-specific deletion of PPAR*γ* results in the complete absence of white adipose tissue in mice [41]. Several transcription factors are known to promote adipocyte development, but none are as important as PPAR*γ*; therefore, this nuclear receptor family member is considered the major regulator of adipogenesis [35]. BJT also downregulated the PPAR*γ* levels in 3T3-L1 adipocytes, epididymal adipose tissue, and liver, suggesting that BJT can block adipocyte development in adipose and liver tissue through the inhibition of PPAR*γ*. The SREBP family of transcription factors modulate cholesterol and fatty acid metabolism; the family comprises of three members, SREBP1a, SREBP1c, and SREBP2 [43]. SREBP1a and SREBP2 are critical for the regulation of target genes involved in cholesterol metabolism, while SREBP1c is related to lipid synthesis [44]. Furthermore, the activation of SREBP1 leads to fatty liver through the stimulation of transcription of the network encompassing, at least in part, the synthesis of fatty acids and triglycerides [45]. Given that the expression of PPAR*γ*, C/EBP*α*, and SREBP1 are the major gene targets for the suppression of adipogenesis in differentiated cells and tissues, the hematoxylin/eosin staining showed that BJT decreased both the enlargement of adipocyte in epididymal adipose tissue and lipid accumulation in liver tissue, which is thought to be correlated with the inhibition of these adipogenic markers in epididymal adipose and liver tissues (Figure 5). Overall, the suppressive effects of BJT on weight gain may be mediated by the downregulation of the expression of adipogenesis-related genes, and the present research provides a partial explanation for the antiadipogenic properties of BJT.

## 5. Conclusions

In the present study, BJT induced weight loss and alleviated lipid accumulation in epididymal adipose and liver tissues by affecting the adipogenic transcription factors: PPAR*γ*, C/EBP*α*, and SREBP1. Further studies are needed to fully elucidate the antiadipogenic properties of BJT.

## Figures and Tables

**Table 1:** Real-time PCR primer sequences.

Gene	Forward (5′-3′)	Reverse (5′-3′)
PPAR*γ*	ATCGAGTGCCGAGTCTGTGG	GCAAGGCACTTCTGAAACCG
SREBP1	ATCGCAAACAAGCTGACCTG	AGATCCAGGTTTGAGGTGGG
C/EBP*α*	GGAACTTGAAGCACAATCGATC	TGGTTTAGCATAGACGTGCACA
GAPDH	GACGGCCGCATCTTCTTGT	CACACCGACCTTCACCATTTT

**Table 2:** Effects of BJT administration on serum biochemical parameters in HFD-induced mice.

Parameters groups	TG (mg/dl)	TC (mg/dl)	ALT (U/l)	AST (U/l)	Creatinine (mg/dl)	BUN (mg/dl)
CON	125.83 ± 10.62	100.39 ± 8.82	31.30 ± 4.96	98.52 ± 19.44	0.20 ± 0.07	21.94 ± 4.04
HFD	179.79 ± 49.46^##^	182.14 ± 31.32^###^	30.53 ± 5.38	93.66 ± 17.49	0.19 ± 0.07	20.16 ± 0.94
BJT	116.95 ± 16.10^∗∗∗^	153.57 ± 8.75^∗^	31.68 ± 6.72	97.34 ± 17.62	0.18 ± 0.07	19.41 ± 2.31

The values are represented as mean ± S.D (*n* = 6). Abbreviations: TG: triglyceride; TC: total cholesterol; ALT: alanine aminotransferase; AST: aspartate aminotransferase; BUN: blood urea nitrogen.

## Data Availability

The datasets used and/or analyzed in this study are available from the corresponding authors upon reasonable request.

## Abbreviations

BJT: Bojungchiseub-tang
HFD: High-fat diet
PPAR*γ*: Peroxisome proliferator-activated receptor *γ*
C/EBP*α*: CCAAT/enhancer-binding protein *α*
SREBP1: Sterol regulatory element-binding protein 1.

## References

[1] Lavie C. J., Pandey A., Lau D. H., Alpert M. A., Sanders P. (2017). Obesity and atrial fibrillation prevalence, pathogenesis, and prognosis: effects of weight loss and exercise. *Journal of the American College of Cardiology*.

[2] Nepali S., Cha J. Y., Ki H. H. (2018). Chrysanthemum indicum inhibits adipogenesis and activates the AMPK pathway in high-fat-diet-induced obese mice. *The American Journal of Chinese Medicine*.

[3] Guo X., Li F., Xu Z. (2017). DOCK2 deficiency mitigates HFD-induced obesity by reducing adipose tissue inflammation and increasing energy expenditure. *Journal of Lipid Research*.

[4] Kusminski C. M., Bickel P. E., Scherer P. E. (2016). Targeting adipose tissue in the treatment of obesity-associated diabetes. *Nature Reviews. Drug Discovery*.

[5] Motomura M., Shimokawa F., Kobayashi T. (2019). Relationships between expression levels of genes related to adipogenesis and adipocyte function in dogs. *Molecular Biology Reports*.

[6] Ghaben A. L., Scherer P. E. (2019). Adipogenesis and metabolic health. *Nature Reviews. Molecular Cell Biology*.

[7] Lakhani H. V., Sharma D., Dodrill M. W. (2018). Phenotypic alteration of hepatocytes in non-alcoholic fatty liver disease. *International Journal of Medical Sciences*.

[8] Kim I. H., Nam T. J. (2017). Enzyme-treated Ecklonia cava extract inhibits adipogenesis through the downregulation of C/EBP*α* in 3T3-L1 adipocytes. *International Journal of Molecular Medicine*.

[9] Wang Z., Kim J. H., Jang Y. S., Kim C. H., Lee J. Y., Lim S. S. (2017). Anti-obesity effect ofSolidago virgaureavar.giganteaextract through regulation of adipogenesis and lipogenesis pathways in high-fat diet-induced obese mice (C57BL/6N). *Food & Nutrition Research*.

[10] Chang E., Kim C. (2019). Natural products and obesity: a focus on the regulation of mitotic clonal expansion during adipogenesis. *Molecules*.

[11] Jun X. (1999). Tongyibaojian Guo Translation Committee. *Translation of Dongyibaojian*.

[12] Wang J., Wang Q., Li L. (2013). Phlegm-dampness constitution: genomics, susceptibility, adjustment and treatment with traditional Chinese medicine. *The American Journal of Chinese Medicine*.

[13] Vasileiou A. M., Bull R., Kitou D., Alexiadou K., Garvie N. J., Coppack S. W. (2011). Oedema in obesity; role of structural lymphatic abnormalities. *International Journal of Obesity*.

[14] Shin S. S., Yoon M. (2018). Korean red ginseng (Panax ginseng) inhibits obesity and improves lipid metabolism in high fat diet-fed castrated mice. *Journal of Ethnopharmacology*.

[15] Song M., Lim S.-K., Wang J.-H., Kim H. (2018). The root of Atractylodes macrocephala Koidzumi prevents obesity and glucose intolerance and increases energy metabolism in mice. *International Journal of Molecular Sciences*.

[16] Kim J. H., Sim H. A., Jung D. Y. (2019). Poria cocus Wolf extract ameliorates hepatic steatosis through regulation of lipid metabolism, inhibition of ER stress, and activation of autophagy via AMPK activation. *International Journal of Molecular Sciences*.

[17] Wang Y., Zhu Y., Ruan K., Wei H., Feng Y. (2014). MDG-1, a polysaccharide from Ophiopogon japonicus, prevents high fat diet-induced obesity and increases energy expenditure in mice. *Carbohydrate Polymers*.

[18] Park I. S., Kim B., Han Y. (2020). Decursin and decursinol angelate suppress adipogenesis through activation of *β*-catenin signaling pathway in human visceral adipose-derived stem cells. *Nutrients*.

[19] Sung Y. Y., Kim D. S., Kim H. K. (2015). Akebia quinata extract exerts anti-obesity and hypolipidemic effects in high-fat diet-fed mice and 3T3-L1 adipocytes. *Journal of Ethnopharmacology*.

[20] Na H. Y., Lee B. C. (2019). Scutellaria baicalensis alleviates insulin resistance in diet-induced obese mice by modulating inflammation. *International Journal of Molecular Sciences*.

[21] Kim I. S., Yang M. R., Lee O. H., Kang S. N. (2011). Antioxidant activities of hot water extracts from various spices. *International Journal of Molecular Sciences*.

[22] Park Y. J., Seo D. W., Ju J. Y., Cha Y. Y., An H. J. (2019). The antiobesity effects of Buginawa in 3T3-L1 preadipocytes and in a mouse model of high-fat diet-induced obesity. *BioMed Research International*.

[23] Park Y. J., Lee G. S., Cheon S. Y., Cha Y. Y., An H. J. (2019). The anti-obesity effects of Tongbi-san in a high-fat diet-induced obese mouse model. *BMC Complementary and Alternative Medicine*.

[24] Ansari A., Bose S., Yadav M. K. (2016). CST, an herbal formula, exerts anti-obesity effects through brain-gut-adipose tissue axis modulation in high-fat diet fed mice. *Molecules*.

[25] Lee S. J., Kim W. I., Kang K. H. (2014). Inhibitory effects of Bojungchiseub-tang on adipocyte differentiation and adipogenesis in 3T3-L1 preadipocytes. *Korean Journal of Oriental Physiology & Pathology*.

[26] Lee H., Kim M., Shik Shin S., Yoon M. (2014). Ginseng treatment reverses obesity and related disorders by inhibiting angiogenesis in female db/db mice. *Journal of Ethnopharmacology*.

[27] Kim J. H., Hahm D. H., Yang D. C., Kim J. H., Lee H. J., Shim I. (2005). Effect of crude saponin of Korean red ginseng on high-fat diet-induced obesity in the rat. *Journal of Pharmacological Sciences*.

[28] Karu N., Reifen R., Kerem Z. (2007). Weight gain reduction in mice fed Panax ginseng saponin, a pancreatic lipase inhibitor. *Journal of Agricultural and Food Chemistry*.

[29] Mollah M. L., Kim G. S., Moon H. K. (2009). Antiobesity effects of wild ginseng (Panax ginseng C.A. Meyer) mediated by PPAR-gamma, GLUT4 and LPL in ob/ob mice. *Phytotherapy Research*.

[30] Lee H., Park D., Yoon M. (2013). Korean red ginseng (Panax ginseng) prevents obesity by inhibiting angiogenesis in high fat diet-induced obese C57BL/6J mice. *Food and Chemical Toxicology*.

[31] Lee S. H., Lee H. J., Lee Y. H. (2012). Korean red ginseng (Panax ginseng) improves insulin sensitivity in high fat fed Sprague-Dawley rats. *Phytotherapy Research*.

[32] Kim C. K., Kim M., Oh S. D. (2011). Effects of Atractylodes macrocephala Koidzumi rhizome on 3T3-L1 adipogenesis and an animal model of obesity. *Journal of Ethnopharmacology*.

[33] Oliveros E., Somers V. K., Sochor O., Goel K., Lopez-Jimenez F. (2014). The concept of normal weight obesity. *Progress in Cardiovascular Diseases*.

[34] Greenway F. L. (2015). Physiological adaptations to weight loss and factors favouring weight regain. *International Journal of Obesity*.

[35] Mota de Sa P., Richard A. J., Hang H., Stephens J. M. (2017). Transcriptional regulation of adipogenesis. *Comprehensive Physiology*.

[36] Kim M. O., Ryu H. W., Choi J. H. (2016). Anti-obesity effects of spiramycin in vitro and in vivo. *PLoS One*.

[37] Lourenco A. R., Coffer P. J. (2017). A tumor suppressor role for C/EBP*α* in solid tumors: more than fat and blood. *Oncogene*.

[38] He Y. F., Liu F. Y., Zhang W. X. (2015). Tangeritin inhibits adipogenesis by down-regulating C/EBP*α*, C/EBP*β*, and PPAR*γ* expression in 3T3-L1 fat cells. *Genetics and Molecular Research*.

[39] Boughanem H., Cabrera-Mulero A., Millan-Gomez M. (2019). Transcriptional analysis of FOXO1, C/EBP-*α* and PPAR-*γ*2 genes and their association with obesity-related insulin resistance. *Genes*.

[40] Lee J. E., Schmidt H., Lai B., Ge K. (2019). Transcriptional and epigenomic regulation of adipogenesis. *Molecular and Cellular Biology*.

[41] Wafer R., Tandon P., Minchin J. E. N. (2017). The role of peroxisome proliferator-activated receptor gamma (PPARG) in adipogenesis: applying knowledge from the fish aquaculture industry to biomedical research. *Frontiers in Endocrinology*.

[42] Hsiao T. J., Lin E. (2015). The Pro12Ala polymorphism in the peroxisome proliferator-activated receptor gamma (PPARG) gene in relation to obesity and metabolic phenotypes in a Taiwanese population. *Endocrine*.

[43] Bengoechea-Alonso M. T., Ericsson J. (2016). The phosphorylation-dependent regulation of nuclear SREBP1 during mitosis links lipid metabolism and cell growth. *Cell Cycle*.

[44] Wang N., Liu Y., Ma Y., Wen D. (2018). Hydroxytyrosol ameliorates insulin resistance by modulating endoplasmic reticulum stress and prevents hepatic steatosis in diet-induced obesity mice. *The Journal of Nutritional Biochemistry*.

[45] Prodanovic R., Koricanac G., Vujanac I. (2016). Obesity-driven prepartal hepatic lipid accumulation in dairy cows is associated with increased CD36 and SREBP-1 expression. *Research in Veterinary Science*.

